# Abortive PDCoV infection triggers Wnt/β-catenin pathway activation, enhancing intestinal stem cell self-renewal and promoting chicken resistance

**DOI:** 10.1128/jvi.00137-25

**Published:** 2025-03-26

**Authors:** Shuai Zhang, Yanan Cao, Yanjie Huang, Xueli Zhang, Chunxiao Mou, Tao Qin, Zhenhai Chen, Wenbin Bao

**Affiliations:** 1 College of Animal Science and Technology, Yangzhou University 614678 , Yangzhou, China; 2 College of Veterinary Medicine, Yangzhou University 614704, Yangzhou, China; Loyola University Chicago - Health Sciences Campus, Maywood, Illinois, USA

**Keywords:** PDCoV, pig, abortive infection, chicken, intestinal stem cells, intestinal organoids, intestinal barrier, Wnt/β-catenin signaling pathway

## Abstract

Porcine deltacoronavirus (PDCoV) is an emerging coronavirus causing economic losses to swine industries worldwide. PDCoV can infect chickens under laboratory conditions, usually with no symptoms or mild symptoms, and may cause outbreaks in backyard poultry and wildfowl, posing a potential risk of significant economic loss to the commercial poultry industry. However, the reasons for such a subdued reaction after infection are not known. Here, using chicken intestinal organoid monolayers, we found that although PDCoV infects them nearly as well as porcine intestinal organoid monolayers, infection did not result in detectable amounts of progeny virus. In *ex vivo* and *in vivo* experiments using chickens, PDCoV infection failed to initiate interferon and inflammatory responses. Additionally, infection did not result in a disrupted intestinal barrier nor a reduced number of goblet cells and mucus secretion, as in pigs. In fact, the number of goblet cells increased as did the secreted mucus, thereby providing an enhanced protective barrier. *Ex vivo* PDCoV infection in chicken triggered activation of the Wnt/β-catenin pathway with the upregulation of Wnt/β-catenin pathway genes (*Wnt3a*, *Lrp5*, *β-catenin*, and *TCF4*) and Wnt target genes (*Lgr5*, *cyclin D1*, and *C-myc*). This activation stimulates the self-renewal of intestinal stem cells (ISCs), accelerating ISC-mediated epithelial regeneration by significant up-regulation of *PCNA* (transiently amplifying cells), *BMI1* (ISCs), and *Lyz* (Paneth cells). Our data demonstrate that abortive infection of PDCoV in chicken cells activates the Wnt/β-catenin pathway, which facilitates the self-renewal and proliferation of ISCs, contributing to chickens’ resistance to PDCoV infection.

**IMPORTANCE:**

The intestinal epithelium is the main target of PDCoV infection and serves as a physical barrier against pathogens. Additionally, ISCs are charged with tissue repair after injury, and promoting rapid self-renewal of intestinal epithelium will help to re-establish the physical barrier and maintain intestinal health. We found that PDCoV infection in chicken intestinal organoid monolayers resulted in abortive infection and failed to produce infectious virions, disrupt the intestinal barrier, reduce the number of goblet cells and mucus secretion, and induce innate immunity, but rather increased goblet cell numbers and mucus secretion. Abortive PDCoV infection activated the Wnt/β-catenin pathway, enhancing ISC renewal and accelerating the renewal and replenishment of shed PDCoV-infected intestinal epithelial cells, thereby enhancing chicken resistance to PDCoV infection. This study provides novel insights into the mechanisms underlying the mild or asymptomatic response to PDCoV infection in chickens, which is critical for understanding the virus’s potential risks to the poultry industry.

## INTRODUCTION

Coronaviruses (CoVs) are enveloped viruses with a positive-sense single-stranded RNA genome and are classified into four genera: *Alphacoronavirus*, *Betacoronavirus*, *Gammacoronavirus*, and *Deltacoronavirus*. CoVs have a broad host range, with *Alphacoronavirus* and *Betacoronavirus* primarily infecting mammals and *Gammacoronavirus* and *Deltacoronavirus* primarily infecting birds (1–4). Just during the 21st century, five novel human and swine CoVs (severe acute respiratory syndrome coronavirus [SARS-CoV], porcine deltacoronavirus [PDCoV], Middle East respiratory syndrome [MERS], swine acute diarrhea syndrome coronavirus [SADS-CoV], and severe acute respiratory syndrome coronavirus 2 [SARS-CoV-2]) have emerged and spread globally, causing significant morbidity, mortality, and economic loss (3, 5–9). CoVs have sporadically crossed a species barrier and infected new hosts, in some cases causing profound challenges globally (3, 4, 10, 11). PDCoV is the only *Deltacoronavirus* that infects mammals; the natural reservoirs of other *Deltacoronaviruses* have mostly been identified in avian species (10, 11).

PDCoV was initially detected in pig feces in Hong Kong in 2012 (3), and its etiologic role in causing diarrhea in its natural host, the pig, was subsequently identified in the United States in 2014 (12). Shortly thereafter, PDCoV spread to many Asian and North American countries, causing significant economic losses to the global swine industry (13, 14). PDCoV has a broad host range, which includes pigs, wild birds, chickens, cattle, and humans (4, 15–17). Phylogenetic and genome-based recombination analysis suggests that PDCoV may have originated from multiple recombination events between different sparrow CoVs (13, 18). The PDCoV genome is closely related to the sparrow CoV HKU17 genome with more than 90% amino acid identity (3, 11). An accumulating body of evidence indicates that PDCoV has cross-species transmission ability and is able to infect poultry. For example, Alhamo et al. and Li et al. (4) report that PDCoV infects chicken cells *in vitro* (4, 19), and Iseki et al. (20) report that PDCoV was efficiently propagated in inoculated embryonated chicken eggs (20). Boley et al. and Liang et al. report that progeny virus was detected in experimentally PDCoV-infected chickens (16, 21). The molecular mechanisms accounting for the interspecies transmission of PDCoV remain largely unknown. Given the enormous potential for interspecies transmission of PDCoV and its potential risk to the livestock and poultry industries, prevention and control of PDCoV is critical.

PDCoV is an enteric virus that mainly infects epithelial cells in the small intestine of pigs, causing acute watery diarrhea, vomiting, and dehydration (8, 12, 22). Older pigs generally recover from infection (23), but the mortality in piglets is high (22). The intestinal epithelium is a physical barrier between host and the intestinal microenvironment. Gut barrier integrity and intestinal homeostasis are the result of constant renewal (every 4–5 days) that is driven by intestinal stem cells (ISCs) (24–26). Lgr5^+^ ISCs, located at the base of the crypt, differentiate into proliferating transiently amplifying (TA) cells. Some of these TA cells migrate upward to the villi, eventually differentiating into absorptive enterocytes or enteroendocrines, goblets, or tuft cells to complete epithelial renewal, while the remaining TA cells migrate downward to the bottom of the crypts and differentiate into Paneth cells to regulate the proliferation and differentiation of ISCs (27–30). The self-renewal and differentiation of ISCs is critical for maintaining or regenerating after damage the integrity of the intestinal epithelium (26, 31, 32). The Wnt/β-catenin signaling pathway plays an essential role in the self-renewal and differentiation of ISCs during a post-injury repair (33–36). The classical Wnt/β-catenin signaling pathway is triggered by secreted Wnt proteins binding to cell surface transmembrane receptors (frizzled and low‐density lipoprotein receptor-related protein 5/6). This interaction leads to inhibition of destruction complexes, including the tumor suppressor axin and adenomatous polyposis coli as well as constitutively active kinases glycogen synthase kinase 3β and casein kinase I. Unphosphorylated β-catenin escapes from proteasomal degradation, translocates to the nucleus, and interacts with the T-cell factor/lymphocyte enhancer factor transcription factors to induce transcription of target genes, including *c-myc*, *cyclin D1*, *Lgr5*, and *matrix metalloproteinase* (37). The activation of the Wnt/β-catenin pathway accelerates ISC repopulation and promotes gut epithelial regeneration. Previous studies have shown that the activation of the Wnt/β-catenin pathway inhibits porcine reproductive and respiratory syndrome virus (PRRSV) infection and HIV transcription (38–40), suggesting that modulation of the Wnt/β-catenin pathway is crucial for virus infection, epithelial regeneration, and repair after infection, although this has not been studied during PDCoV infection.

In this study, using the natural host of PDCoV (pigs) as positive control, we investigated the susceptibility of chickens to PDCoV infection. Firstly, we evaluated the PDCoV infection, anti-viral response, inflammatory response, and intestinal barrier integrity of infected intestinal organoid monolayers. Subsequently, we investigated the effects of PDCoV infection on Wnt/β-catenin pathway-related genes and ISC development. Finally, we examined the susceptibility of chickens to PDCoV infection, as well as the tropism and pathogenicity of PDCoV in small intestinal tissues of chickens. Our data demonstrated that PDCoV infection in chicken intestinal organoid monolayers resulted in abortive infection and failure to produce infectious virions and induced innate immunity. Furthermore, PDCoV infection did activate the Wnt/β-catenin signaling pathway, resulting in increased ISC renewal, and accelerated renewal and replenishing of shed PDCoV-infected intestinal epithelial cells, thereby enhancing chicken resistance to PDCoV infection.

## RESULTS

### Isolation and comparison of chicken and porcine intestinal organoids and organoid monolayers

**Fig 1:**
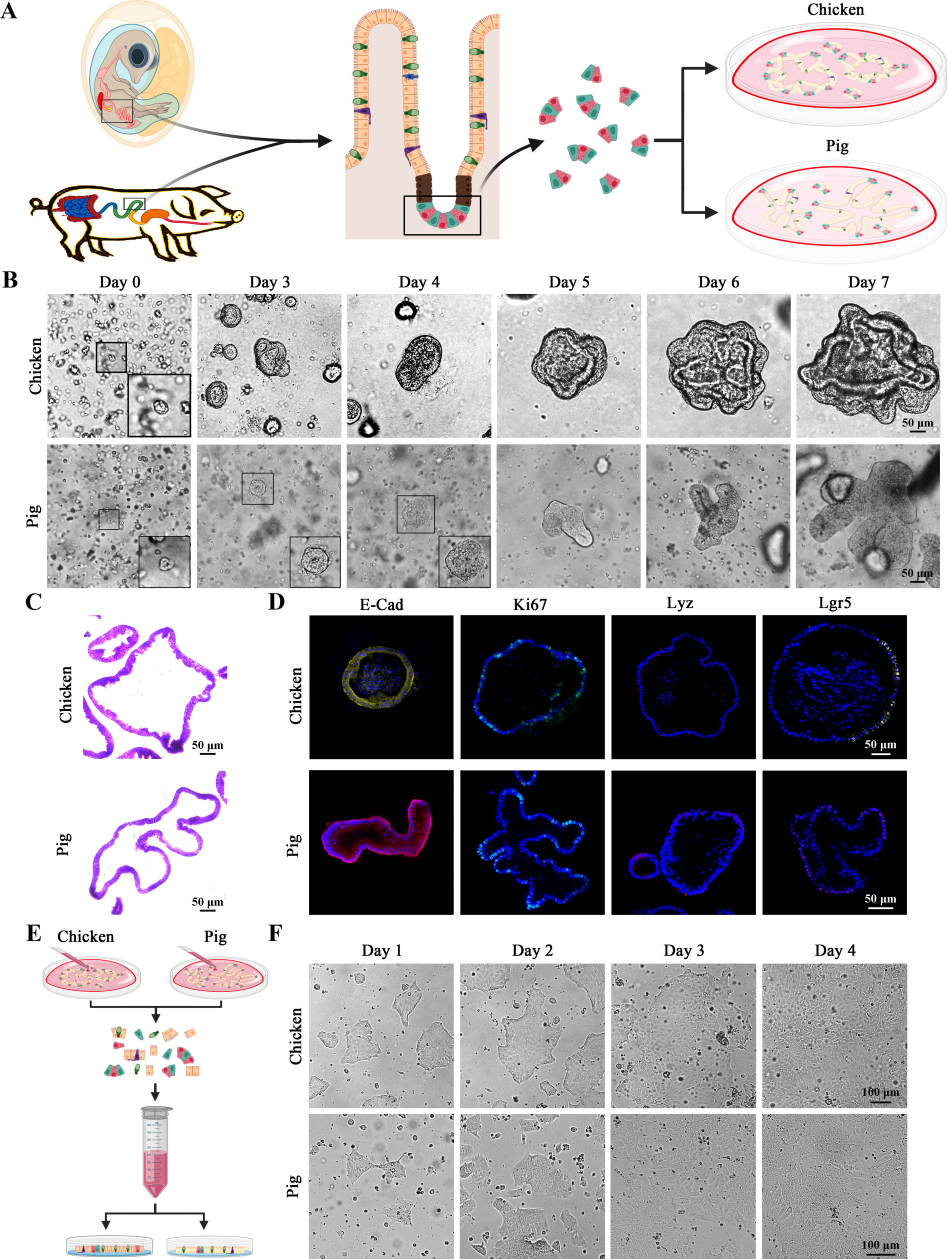
Isolation and comparison of chicken and pig intestinal 3D organoids and 2D organoid monolayers. (A) Schematic diagram illustrating intestinal organoid isolation and culture. (B) Time course of development of chicken and pig 3D intestinal organoids as observed by light microscopy. Scale bar = 50 µm. (C) Hematoxylin and eosin staining of chicken (upper) and pig (lower) intestinal organoids. Scale bar = 50 µm. (D) Intestinal organoids were subjected to immunofluorescence assay staining for enterocytes (E-Cad), proliferating cells (Ki67), Paneth cells (Lyz), and intestinal stem cells (Lgr5). Scale bar = 50 µm. (E) Schematic diagram of establishment of 2D intestinal organoid monolayers. (F) The dissociated chicken and pig organoids grow into a confluent intestinal organoid monolayer after 4 days. Scale bar = 100 µm.

Schematic of the isolation and culture of chicken- and pig-derived intestinal crypts from intestinal tissues of 18-day-old chicken embryos and jejunal tissues of newborn piglets, respectively (Fig. 1A). The crypts were seeded into Matrigel, and the size of organoids was followed over 7 days (Fig. 1B). Chicken-derived intestinal organoids developed into comparatively larger three-dimensional (3D) structures with more buds; pig-derived intestinal organoids had fewer and elongated buds (Fig. 1B and C). Immunofluorescence staining revealed that both chicken and pig-derived intestinal organoids contained Lgr5^+^ intestinal stem cells and differentiated epithelial cells, including enterocyte (E-Cad), proliferating cells (Ki67), and Paneth cells (lyz) (Fig. 1D).

Generally, most enteric pathogens attack the epithelial cell layer via the apical cell surface. However, the apical membrane of 3D intestinal organoids faces the inside, making it problematic for PDCoV to infect them. To investigate PDCoV infection, we thus sought to develop the intestinal organoid monolayer model through dissociating 3D intestinal organoids into single cells and culturing in microplates (Fig. 1E). Subsequently, cells expanded and differentiated to form a fused intestinal organoid monolayer after 4 days of culturing (Fig. 1F). These results show that we successfully developed chicken and porcine intestinal organoids and organoid monolayers.

### Chicken intestinal organoid monolayers are susceptible to PDCoV infection, but progeny viruses are not produced

**Fig 2:**
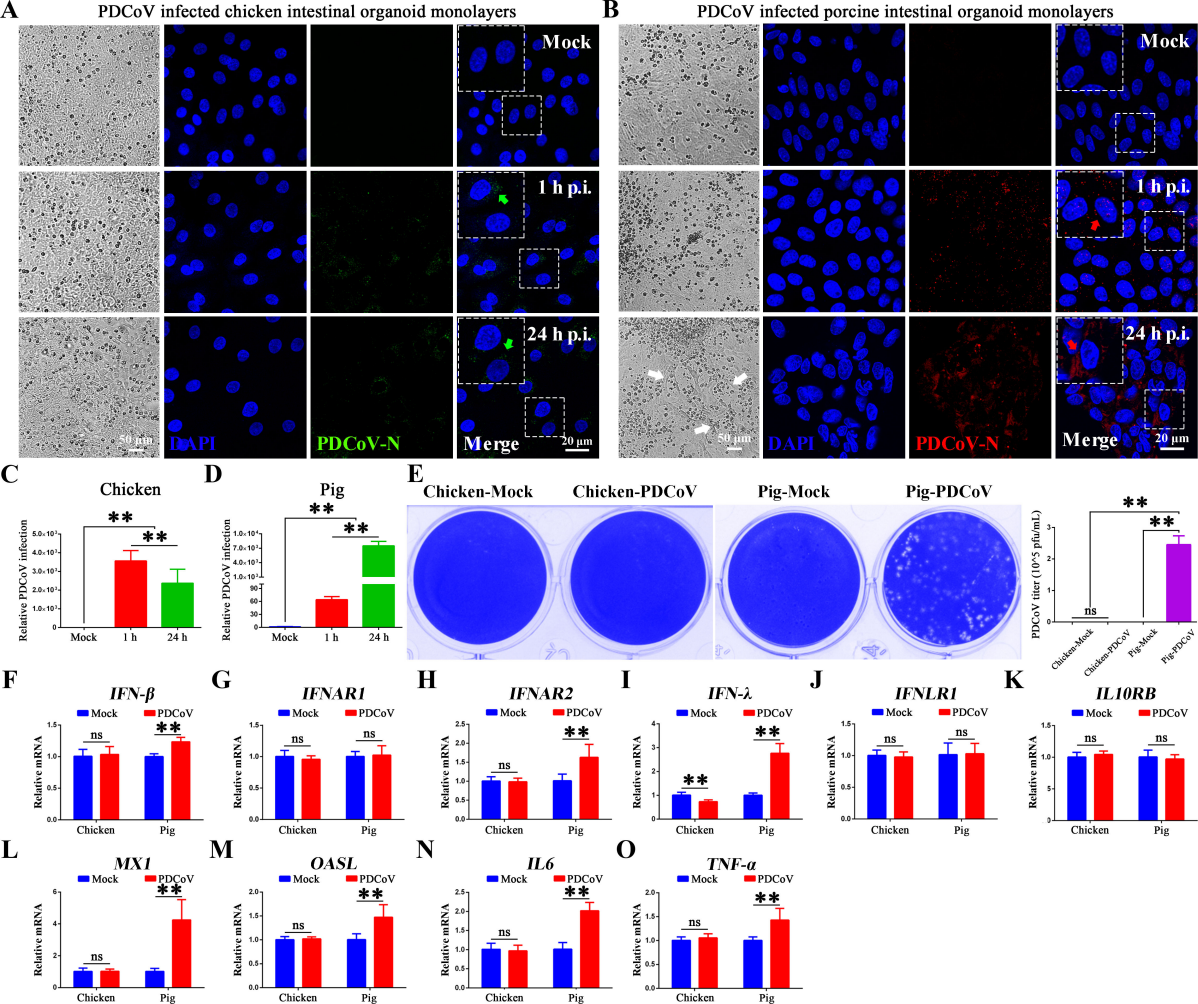
Chicken intestinal organoid monolayers are susceptible to PDCoV infection but do not support replication. (A) Chicken and (B) pig intestinal 2D organoid monolayers were infected with PDCoV (multiplicity of infection [MOI] = 5) for 1 and 24 h. PDCoV-N protein appears green or red, and nuclei appear blue in IFA; cytopathic effect (CPE) is seen by light microscopy in infected porcine organoid monolayers (white arrows) but not in infected chicken organoid monolayers. Scale bar = 20 µm. Viral replication in (C) chicken and (D) pig intestinal 2D monolayer organoids was quantified by RT-qPCR at 1 and 24 h post-infection (MOI = 0.1). (E) Culture supernatants from PDCoV-infected chicken and porcine organoid monolayers at 24 hpi were titered by plaque assay in ST cells. (F–O) mRNA levels of interferons and their respective cellular receptors (*IFN-β*, *IFNAR1*, *IFNAR2*, *IFN-λ*, *IFNLR1*, and *IL10RB*), interferon-stimulated genes (*MX1* and *OASL*), and proinflammatory factors (*IL6* and *TNF-α*) from PDCoV-infected chicken and pig enteroids at 24 hpi. ** *P* < 0.01. ns, not significant.

To evaluate the differences in susceptibility to PDCoV infection, chicken- and pig-derived intestinal organoid monolayers were infected with PDCoV for 1 and 24 h. PDCoV-N-positive signals were detected in both chicken- and pig-derived intestinal organoid monolayers (Fig. 2A and B), although a higher proportion of the pig cells were PDCoV positive, particularly those infected for 24 h (Fig. 2B). Additionally, PDCoV infection resulted in a significant cytopathic effect in pig but not chicken intestinal organoid monolayers (Fig. 2A and B). The kinetics of PDCoV infection in chicken and pig intestinal organoid monolayers were determined by reverse transcription-quantitative real-time PCR (RT-qPCR) (Fig. 2C and D). Consistent with the immunofluorescence assay (IFA) results, PDCoV-infected chicken organoid monolayers exhibited higher levels of PDCoV-N mRNA and more PDCoV-positive cells at 1 h compared to 24 h (Fig. 2A and C), with the level of viral infection declining over time (Fig. S1A). In contrast, PDCoV displayed more efficient replication in porcine organoid monolayers, with PDCoV-N mRNA levels increasing over time (Fig. 2B and D). Levels of infectious progeny virus in the 24 h supernatants of these cells were determined by plaque assay on ST cells. Figure. 2E illustrates that viable viruses were only detected in PDCoV-infected porcine intestinal organoid monolayer supernatants. We next determined the anti-viral response of the organoid monolayers after PDCoV infection. Transcription of *IFN-β* and *IFN-λ* (interferons), *MX1* and *OASL* (interferon-stimulated genes), and *IL6* and *TNF-α* (proinflammatory factors) was significantly induced in PDCoV-infected porcine intestinal organoid monolayers. Levels of *IFNAR2* were also significantly increased, but there was no significant change in other type I and type III interferon receptor mRNA levels. In contrast, except for a significant decrease in *IFN-λ*, there were no significant changes in the levels of these genes in PDCoV-infected chicken intestinal organoid monolayers (Fig. 2F through O). In addition, except for a significant decrease in *IFN-β* at 1 and 12 hpi, transcription of *IFN-λ* (interferon), *MX1* and *OASL* (interferon-stimulated genes), and *IL6* and *TNF-α* (proinflammatory factors) was increased to different degrees in early viral infections (such as 1, 6, and 12 h post-infection). However, mRNA levels of type I (*IFNAR1* and *IFNAR2*) and type III (*IFNLR1* and *IL10RB*) interferon receptors remained unchanged during PDCoV infection (Fig. S1B through K). These data suggested that chicken intestinal organoid monolayers are susceptible to PDCoV infection but are not permitted to replicate and produce progeny viruses from PDCoV.

### PDCoV infection disrupts the intestinal barrier of porcine but not chicken organoid monolayers

**Fig 3:**
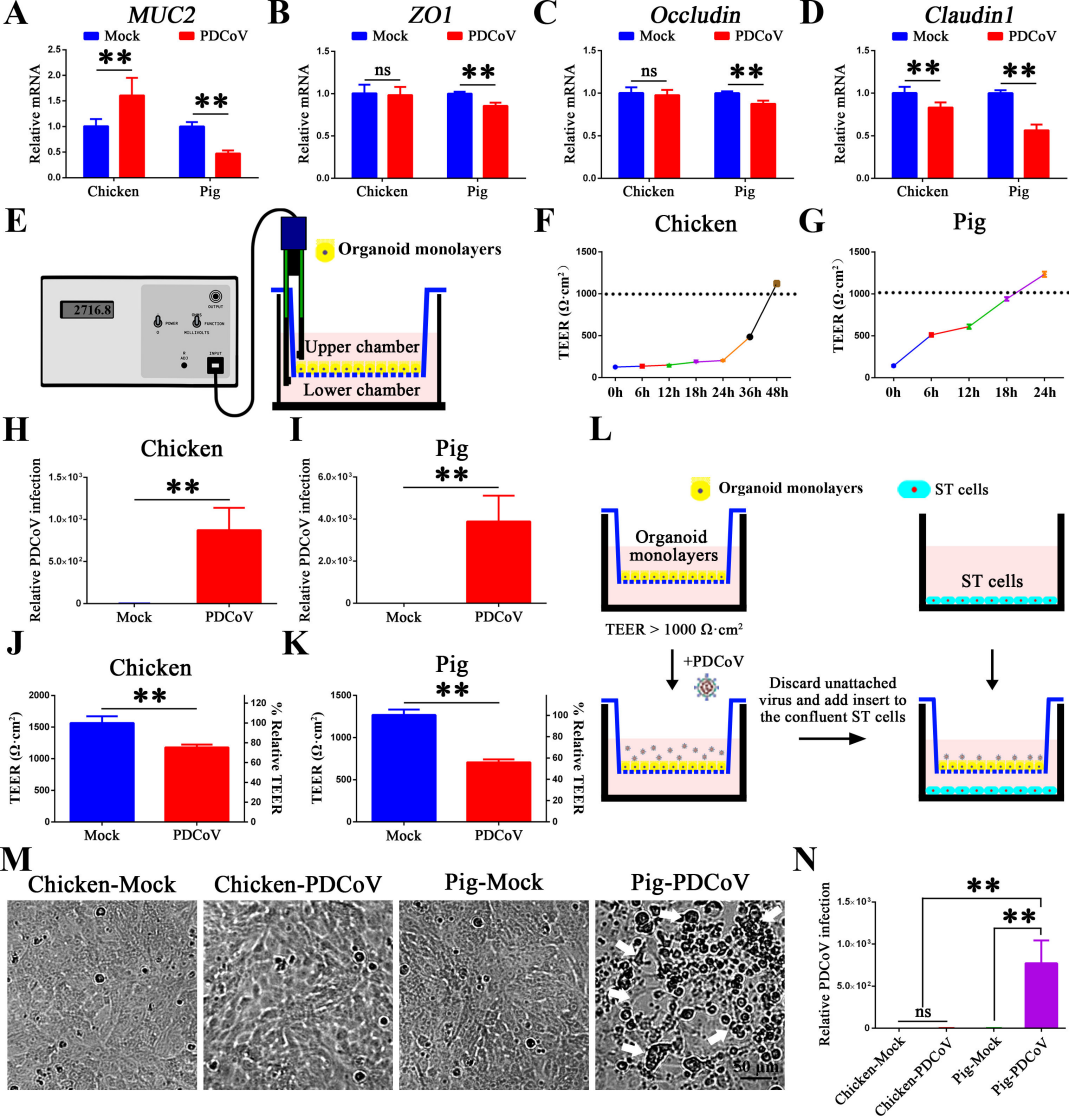
Effect of PDCoV infection on the intestinal barrier integrity of chicken and pig intestinal organoid monolayers. (A–D) mRNA levels of goblet cell-secreted mucin (*MUC2*) and tight junction proteins (*ZO1*, *occludin*, and *claudin-*1) from PDCoV-infected chicken and pig intestinal organoid monolayers. (E) Illustration of the measurement of electrical resistance across the organoid monolayers in Transwell plates. TEER of (F) chicken and (G) porcine organoid monolayers over 48 h. Viral replication in (H) chicken and (I) pig intestinal organoid monolayers was quantified by RT-qPCR at 24 hpi. (MOI = 0.1). TEER values were calculated from PDCoV-infected (J) chicken and (K) pig intestinal organoid monolayers. (L) Schematic of the Transwell co-culture system. (M) Cytopathic effect of PDCoV-infected ST cells (in the lower chamber) as observed by light microscope (white arrows). Scale bar = 50 µm. (N) Viral replication in ST cells from panel M was quantified by RT-qPCR. **P < 0.01. ns, not significant.

The mRNA levels of goblet cell-secreted mucin (*MUC2*) and tight junction proteins (*ZO-1*, *occludin*, and *claudin-*1) were determined by RT-qPCR. The results (Fig. 3A through D) showed that expression of *MUC2*, *ZO1*, *occludin*, and *claudin-*1 was significantly decreased in infected porcine intestinal organoid monolayers. In infected chicken intestinal organoid monolayers, the levels of *MUC2* were significantly increased, and *claudin-1* significantly decreased, while levels of *ZO1* and *occludin* were not significantly different from mock. Fig. 3E illustrates the method used to measure the transepithelial electrical resistance (TEER), an important indicator of intestinal epithelial integrity and permeability (41). The resistance initially increased with time and then reached a stable plateau at 60 h (3,204.9–3,853.8 Ω·cm^2^) (Fig. S2). As shown in Fig. 3F and G, both chicken and pig intestinal organoid monolayers form an intact barrier with TEER over 1,000 Ω·cm^2^ (indicating an intact and tightly connected monolayer), and only those monolayers possessing TEER values higher than 1,000 Ω·cm^2^ were used for PDCoV infection. We then measured the changes in TEER in PDCoV-infected chicken and pig enteroid monolayers and found that (consistent with the results in Fig. 2A through D) PDCoV infects both chicken and porcine organoid monolayers but is more severe in pigs at 24 h (Fig. 3H and I). The TEER decreased by 20% from mock values in chicken organoid monolayers and by 40% in porcine organoid monolayers (Fig. 3J and K). Fig. 3L illustrates the organoid monolayer/ST cell co-culture system used to evaluate the effect of PDCoV infection on intestinal permeability, with permeability being determined by the level of PDCoV infection of the ST cells. Consistently, no cytopathic effect was observed, and no virus was detected in ST cells co-cultured with mock- and PDCoV-infected chicken organoid monolayers (Fig. 3M and N). In PDCoV-infected porcine organoid monolayers, a cytopathic effect was obvious, and PDCoV transcripts were detected in ST cells (Fig. 3M and N). These data demonstrate that PDCoV infects chicken organoid monolayers but does not disrupt their barrier integrity to the same extent that it disrupts the barrier integrity of porcine organoid monolayers.

### PDCoV infection activates the Wnt/β-catenin signaling pathway in chicken organoid monolayers

**Fig 4:**
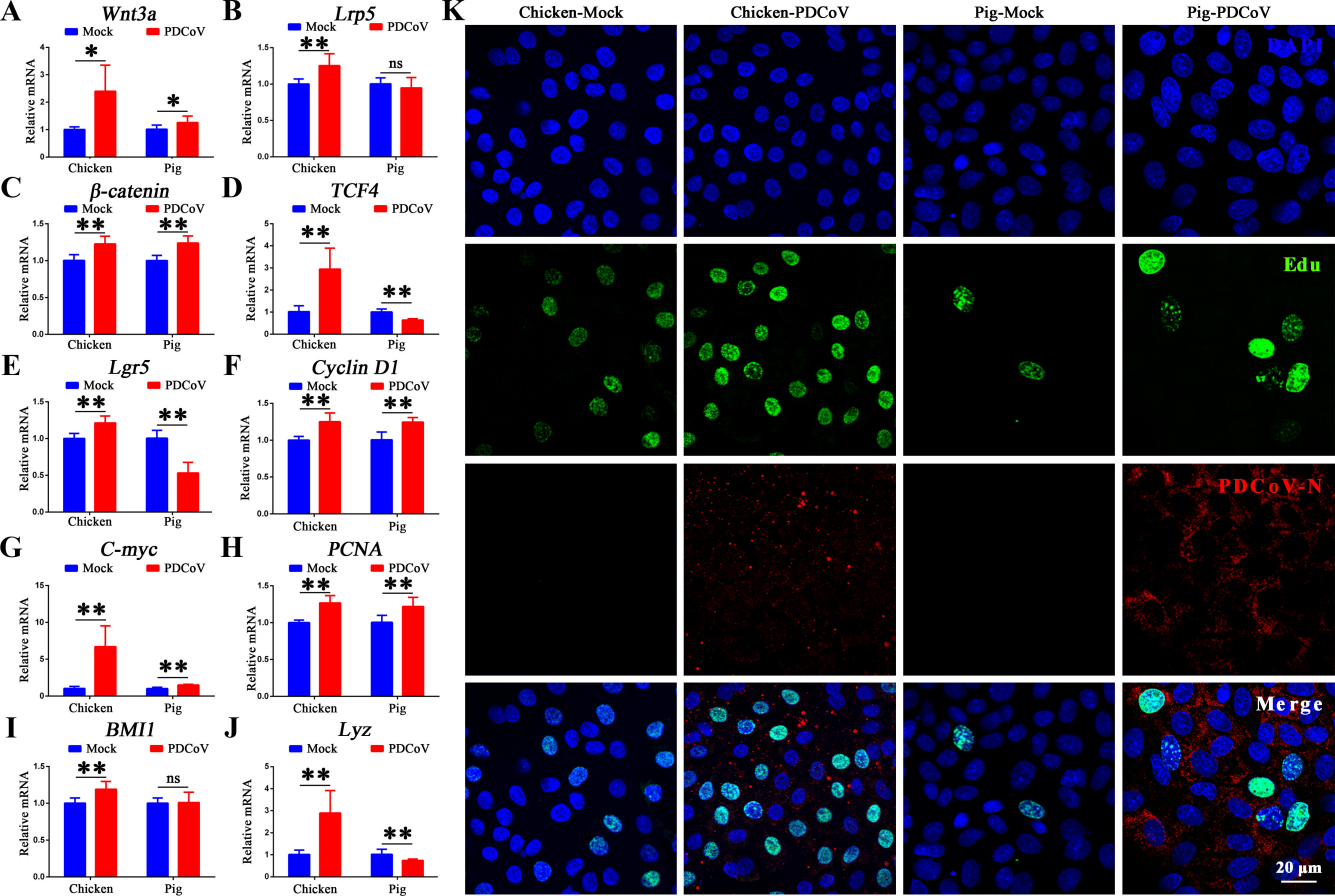
PDCoV infection activates the Wnt/β-catenin signaling pathway, promoting the self-renewal of chicken intestinal stem cells *ex vivo*. (A–J) Levels of *Wnt3a*, *Lrp5*, *β-catenin*, *TCF4*, *Lgr5*, *Cyclin D1*, *c-myc*, *PCNA*, *BMI1*, and *Lyz* from mock- and PDCoV-infected chicken and pig intestinal organoid monolayers at 24 hpi. (K) IFA at 24 hpi of PDCoV-infected chicken and pig intestinal organoid monolayers. Scale bar = 20 µm. *P < 0.05, **P < 0.01. ns, not significant.

After injury, ISCs play a pivotal role in renewal and regeneration of the intestinal epithelium. Notably, the Wnt/β-catenin signaling pathway is indispensable for the proliferation and differentiation of ISCs (42). Hence, we speculated that the activation of the Wnt/β-catenin pathway by PDCoV infection was the reason for the minimal effect of infection on chicken intestinal barrier integrity. To test the hypothesis, we quantified Wnt/β-catenin pathway-related genes by RT-qPCR in infected chicken and porcine organoid monolayers. As illustrated in Fig. 4A through G, Wnt/β-catenin pathway genes (*Wnt3a*, *Lrp5*, *β-catenin*, and *TCF4*) and Wnt target genes (*Lgr5*, *cyclin D1*, and *c-myc*) were significantly upregulated in PDCoV-infected chicken organoid monolayers. The mRNA levels of *PCNA* (TA cells), *BMI1* (ISCs), and *Lyz* (Paneth cells) were also significantly upregulated (Fig. 4H through J). In PDCoV-infected porcine organoid monolayers, *Wnt3a*, *β-catenin*, *cyclin D1*, *c-myc*, and *PCNA* were also elevated, while *TCF4*, *Lgr5*, and *Lyz* were decreased. IFA of organoid monolayers from both chicken and pig revealed that PDCoV infection resulted in about double the number of EdU-positive ISCs (Fig. 4K). The proportion of EdU-positive chicken ISCs from the PDCoV-infected group was 44.6%, while in pig, the proportion was 17.1%. These results indicated that PDCoV infection activates the Wnt/β-catenin signaling pathway, which, in turn, promotes the self-renewal and proliferation of chicken intestinal stem cells, contributing to the renewal and regeneration of the intestinal epithelium after injury.

### PDCoV does not establish productive infection in chickens

**Fig 5:**
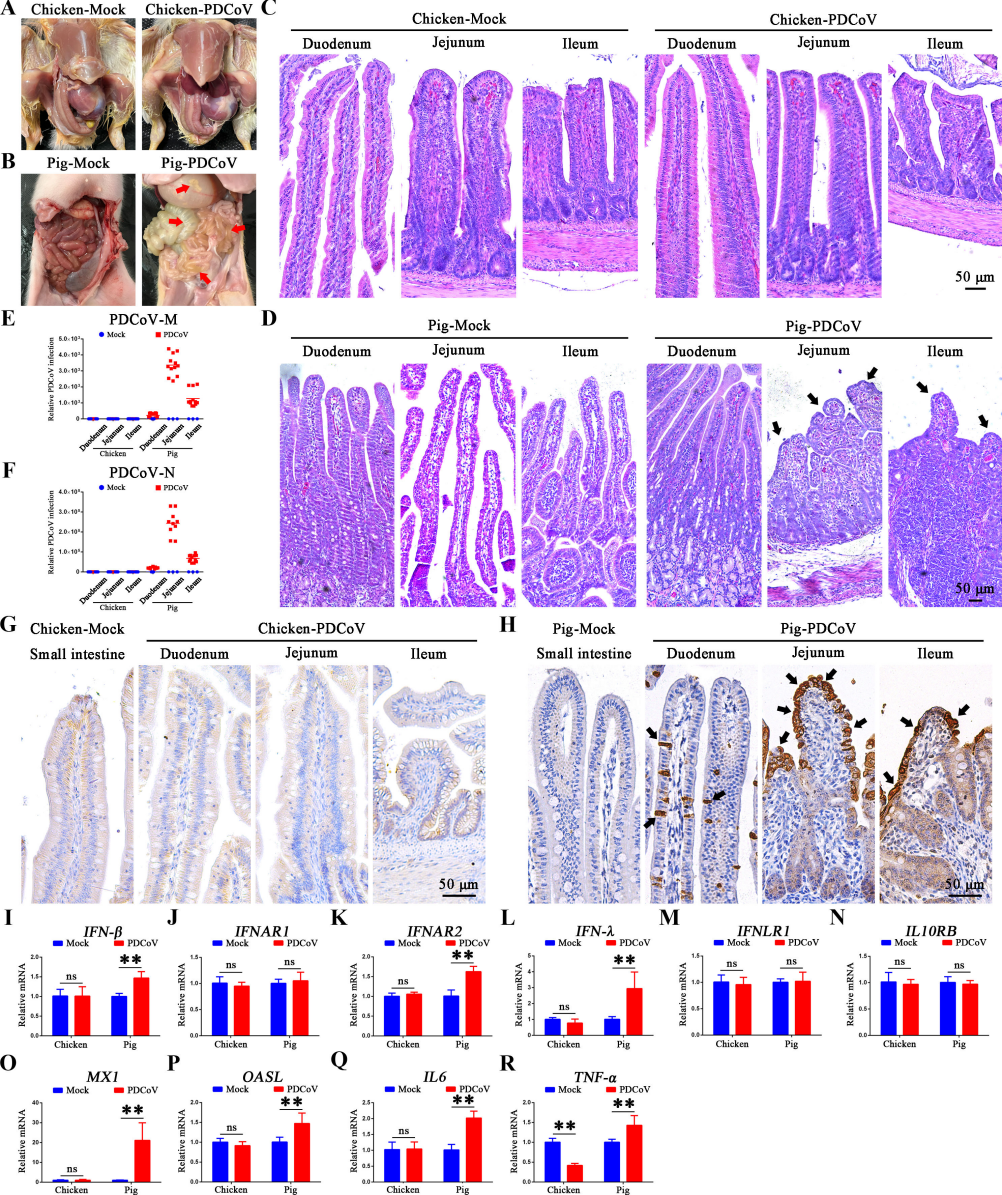
No obvious symptoms or pathological changes were observed in PDCoV-infected chickens compared to infected pigs. Necropsy of mock- and PDCoV-infected (A) chicken and (B) pig. The red arrows indicate intestinal bloating and thinning of the intestinal wall with accumulation of yellow fluid. H&E staining of small intestinal segments from uninfected and infected (C) chicken and (D) pig. The black arrows indicate severe pathological changes in the villus. Scale bar = 50 µm. mRNA levels of (E) PDCoV-M and (F) PDCoV-N in the small intestinal segments of mock- and PDCoV-infected chickens and piglets. Distribution of PDCoV in the small intestinal segments of mock- and PDCoV-infected (G) chicken and (H) pig. PDCoV-N protein stained deep yellow-brown, and the black arrows indicate large amounts of PDCoV-N protein staining in PDCoV-infected pig. Scale bar = 50 µm. (I–R) mRNA levels of interferons and their respective cellular receptors (*IFN-β*, *IFNAR1*, *IFNAR2*, *IFN-λ*, *IFNLR1*, and *IL10RB*), interferon-stimulated genes (*MX1* and *OASL*), and proinflammatory factors (*IL6* and *TNF-α*) from the jejunum of mock- and PDCoV-infected chickens and pigs. **P < 0.01. ns, not significant.

Given that chicken organoid monolayers are susceptible to PDCoV infection but not permissive for virus replication, we carried out a comparative PDCoV challenge experiment in chickens and piglets. All chickens and piglets were sacrificed at 48 hpi when the infected piglets developed severe watery diarrhea and vomiting. It should be noted that at 48 hpi, infected chickens and all animals in the mock infected groups were in good spirits, showing no clinical signs of infection. By necropsy, infected piglets, but not chickens, exhibited obvious clinical lesions (Fig. 5A and B). Hematoxylin and eosin (H&E) staining revealed no histopathological changes in the small intestinal tissue of infected chickens, while in pig jejunum and ileum tissues, villus height was dramatically shortened (Fig. 5C and D). Viral mRNA in the intestinal tissues was quantified by RT-qPCR (Fig. 5E and F). We found no detectable vRNA in the small intestine tissues of PDCoV-challenged chickens but a significant quantity of PDCoV-M and N mRNA in challenged piglets. By immunohistochemistry, we also found no PDCoV-N-positive signal in the small intestine of PDCoV-challenged chickens (Fig. 5G) but a high level of signal in the cytoplasm of intestinal epithelial cells of the jejunum of infected piglets (Fig. 5H). Transcriptional levels of interferons, interferon-stimulated genes, and inflammatory factors in jejunum were evaluated by RT-qPCR. We found that the mRNA levels of IFN and ISG genes in the jejunum tissues of infected piglets were significantly higher than those in uninfected piglets. Consistent with *ex vivo* observations (Fig. 2F through O), PDCoV infection did not impact the levels of these genes in chicken but did result in the inhibition of *TNF-α* (Fig. 5I through R). These results indicated that, in chicken, PDCoV was unable to establish productive infection and did not trigger the anti-viral innate immune responses.

### PDCoV infection promotes the self-renewal and proliferation of chicken intestinal stem cells via the Wnt/β-catenin pathway *in vivo*

**Fig 6:**
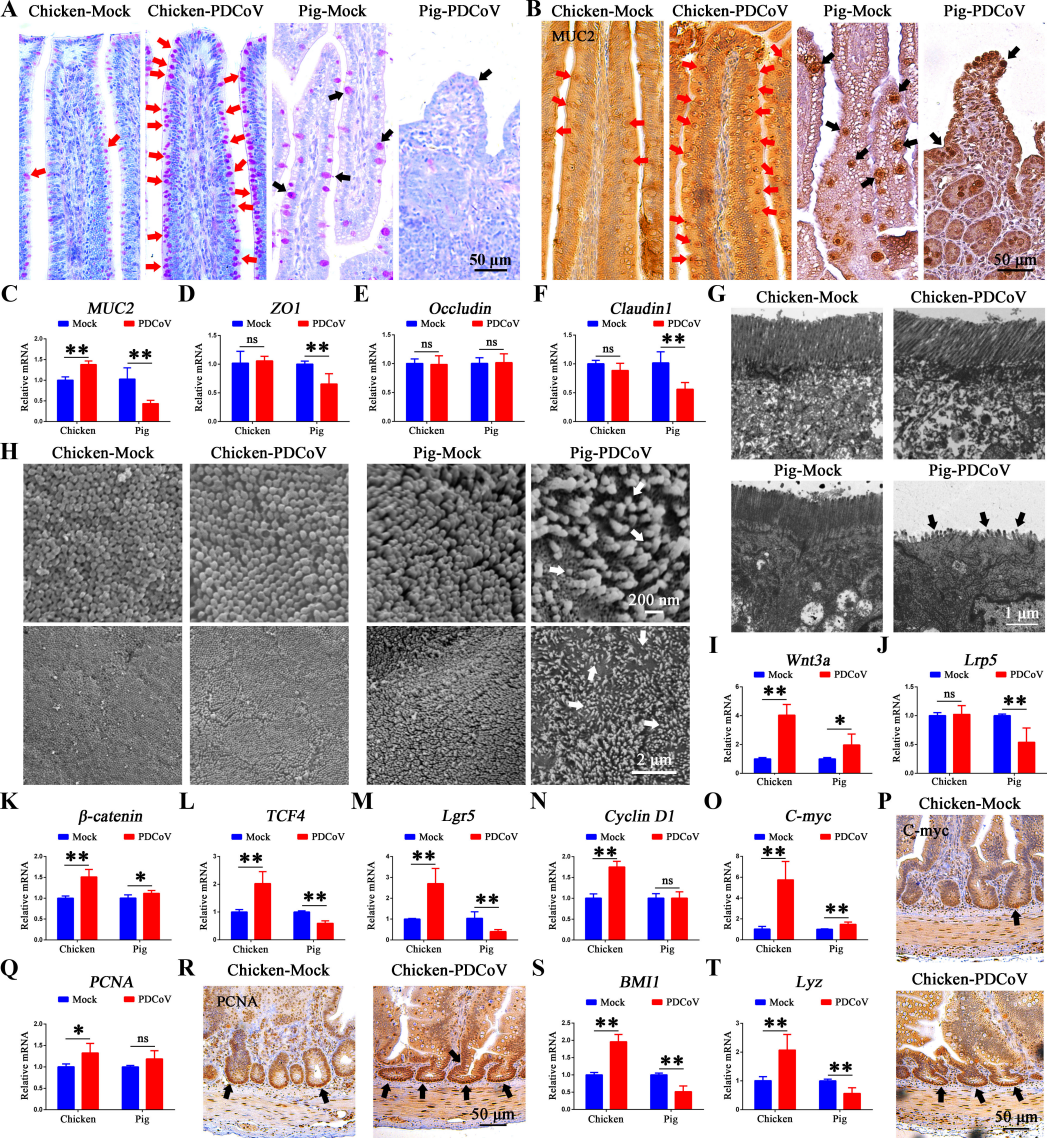
PDCoV infection promotes the self-renewal and proliferation of chicken intestinal stem cells via the Wnt/β-catenin pathway *in vivo*. Jejunum tissues from chickens and piglets were stained for (A) PAS and (B) MUC2. Scale bar = 50 µm. (C–F) mRNA levels of *MUC2* (intestinal mucosal barrier) and *ZO1*, *occludin*, and *claudin-1* (physical barrier) from jejunum tissues of mock- and PDCoV-infected chickens and piglets. (G) Morphology of microvilli as observed by transmission electron microscopy. Scale bar = 1 µm. (H) Morphology of jejunum microvilli as observed by scanning electron microscopy. Scale bar = 200 nm or 1 µm. (I–O) mRNA levels of *Wnt3a*, *Lrp5*, *β-catenin*, *TCF4*, *Lgr5*, *cyclin D1*, and *c-myc* from the jejunum of mock- and PDCoV-infected chicken and pig. (P) Jejunum tissues from mock- and PDCoV-infected chickens were stained for c-myc by immunochemistry (IHC). Scale bar = 50 µm. (Q) *PCNA* levels in homogenized jejunum tissues. (R) Jejunum tissues from mock- and PDCoV-infected chickens were stained by IHC for PCNA. Scale bar = 50 µm. (S and T) mRNA levels of *BMI1* and *Lyz* in homogenized jejunum tissues. *P < 0.05, **P < 0.01. ns, not significant.

We next compared the effect of PDCoV infection on the mucus layer and tight junctions in the jejunum of chickens and pigs. The mucus layer is composed of various mucins (although mainly MUC2) secreted by goblet cells which are usually visualized with periodic acid-Schiff (PAS) staining. Jejunum tissues stained for PAS and immunohistochemistry analysis revealed that the population of goblet cells and their secreted MUC2 were significantly increased in PDCoV-infected chickens, whereas they were significantly decreased in infected piglets (Fig. 6A and B). The mRNA levels of *MUC2* from the jejunum tissues revealed similar results (Fig. 6C). From the homogenized jejunum tissues, we found that PDCoV infection resulted in no significant change in the mRNA levels of tight junction proteins (*ZO-1*, *occludin*, and *claudin-1*) in infected chickens, whereas *ZO-1* and *claudin-1* were significantly decreased in infected piglets (Fig. 6D through F). Ultrastructural analysis of the microvilli on intestinal epithelial cells showed the microvilli of infected chickens were intact and arranged neatly without damage, whereas the microvilli of infected piglets appeared to be severely damaged and shed (Fig. 6G and H). These results indicated that PDCoV infection did not damage the intestinal physical barrier of chickens.

As previously demonstrated *ex vitro*, abortive infection stimulates self-renewal of ISCs and regeneration of epithelium from PDCoV-infected chicken organoid monolayers. We next tested whether ISC self-renewal and differentiation caused by PDCoV infection are triggered via the activation of the Wnt/β-catenin pathway *in vivo*. mRNA levels of *Wnt3a*, *Lrp5*, *β-catenin*, and *TCF4* (Wnt/β-catenin pathway genes) and *Lgr5*, *cyclin D1*, and *c-myc* (Wnt target genes) were quantitated by RT-qPCR. Except for *Lrp5*, these genes were significantly upregulated in the jejunum of PDCoV-infected chicken (Fig. 6I through O). Immunohistochemistry staining chicken jejunum showed PDCoV infection resulted in increased levels of c-myc in the crypt area (Fig. 6P). PDCoV infection also resulted in increased levels of *PCNA* (TA cells) and a higher number of PCNA-positive cells (Fig. 6Q and R), as well as significantly increased *BMI1* (ISCs) and *Lyz* (Paneth cells) (Fig. 6T). In the jejunum of PDCoV-infected pig, levels of *Wnt3a*, *β-catenin*, and *c-myc* were also elevated, but the levels of *Lrp5*, *TCF4*, *Lgr5*, *BMI1*, and *Lyz* were decreased (Fig. 6I through T). Collectively, these data suggested that PDCoV infection activates the Wnt/β-catenin pathway and promotes self-renewal and proliferation of chicken intestinal stem cells, which contributes to the renewal and regeneration of the intestinal epithelium post-infection.

### The Wnt/β-catenin signaling pathway promotes the renewal and regeneration of the intestinal epithelium post-PDCoV infection *ex vivo*

**Fig 7:**
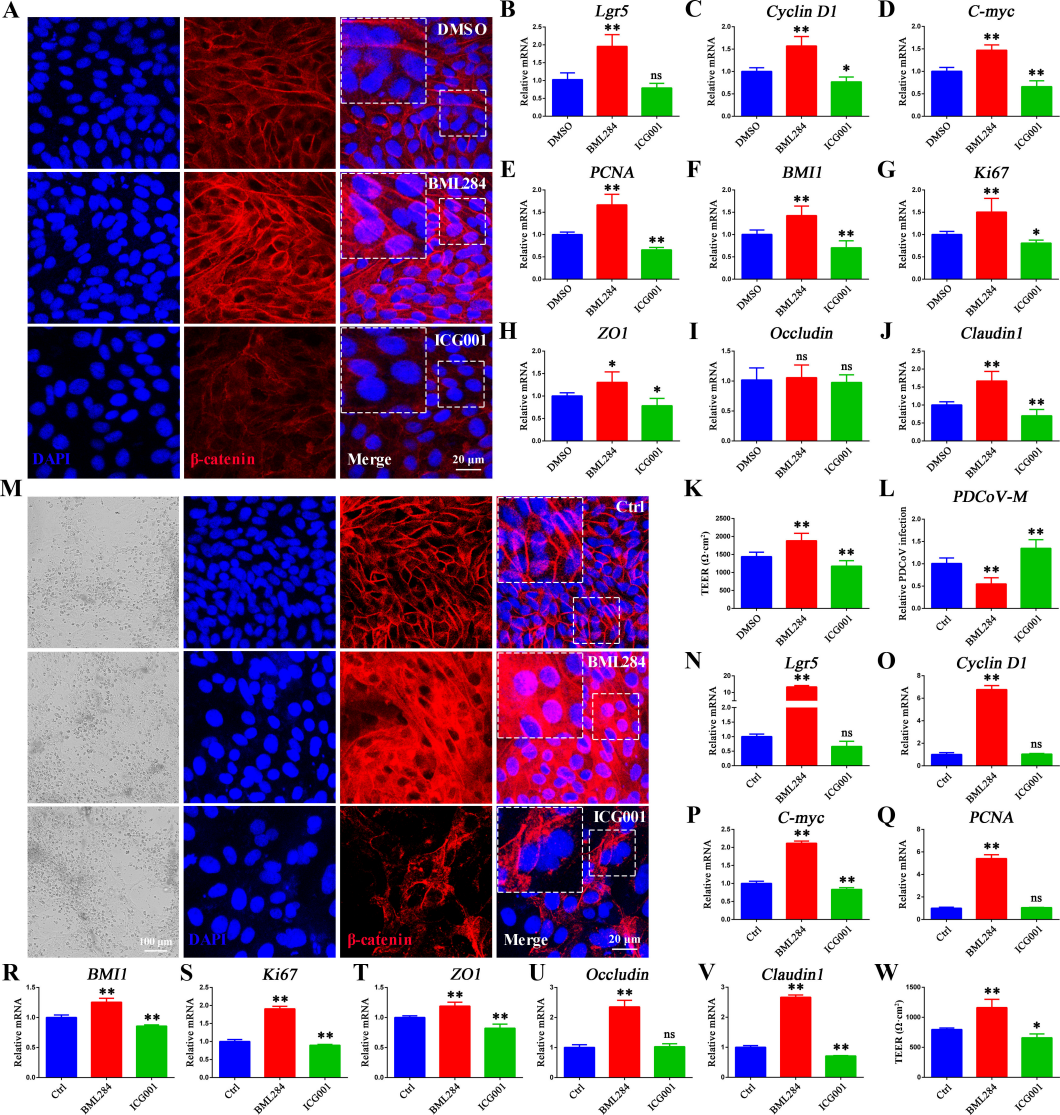
The Wnt/β-catenin pathway promotes the renewal and regeneration of the intestinal epithelium post-PDCoV infection. Porcine intestinal organoid monolayers were treated with 10 µM BML284 or ICG001 for 24 h. (A) The nuclear translocation of β-catenin was observed in organoid monolayer by confocal microscopy. Scale bar = 20 µm. (B–J) mRNA levels of *Lgr5*, *Cyclin D1*, *c-Myc*, *PCNA*, *BMI1*, *Ki67*, *ZO-1*, *occludin*, and *claudin-1* in porcine intestinal organoid monolayers treated with dimethyl sulfoxide (DMSO), BML284, or ICG001 for 24 h. (K) TEER of BML284 or ICG001 in porcine intestinal organoid monolayers treated with DMSO, BML284, or ICG001 for 24 h. (L) mRNA levels of PDCoV-M in porcine intestinal organoid monolayers were treated with BML284 or ICG001 for 24 h then infected with PDCoV for 1 h. (M) The nuclear translocation of β-catenin was observed in organoid monolayer by confocal microscopy. Scale bar = 20 µm. (N–V) mRNA levels of *Lgr5*, *Cyclin D1*, *c-Myc*, *PCNA*, *BMI1*, *Ki67*, *ZO-1*, *occludin*, and *claudin-1* in porcine intestinal organoid monolayers infected with PDCoV for 24 h after 24 h pretreatment with DMSO, BML284, or ICG001. (W) TEER of BML284 or ICG001 pretreated pig intestinal organoid monolayers infected with PDCoV for 24 h. *P < 0.05, **P < 0.01. ns, not significant.

The results described above have demonstrated that PDCoV infection activates the Wnt/β-catenin signaling pathway in chicken organoid monolayers and chickens; thus, we supposed that the Wnt/β-catenin activation contributes to the quick recovery of chickens from PDCoV infection. To further investigate whether activation of Wnt/β-catenin acts to replenish shed PDCoV-infected intestinal epithelial cells and aid repair of a damaged intestinal barrier, we treated porcine organoid monolayers in the Transwell system with 10 µM BML284 (a small molecule activator of the Wnt/β-catenin pathway) or 10 µM ICG001 (an inhibitor of the Wnt/β-catenin pathway). Activation of the Wnt/β-catenin pathway depends on the accumulation of β-catenin, which initiates the transcription of Wnt targets. IFA results showed the accumulation of β-catenin in pig intestinal organoid monolayers treated with BML284 (Fig. 7A). Simultaneously, expression of the Wnt target genes (*Lgr5*, *cyclin D1*, and *c-Myc*) and *BMI1* was significantly increased as were mRNA levels of proliferation-related genes including *PCNA* and *Ki67*. Accordingly, ICG001 treatment resulted in reduced accumulation of β-catenin and inhibition of the Wnt/β-catenin-related genes (Fig. 7B through G). Subsequently, the epithelial integrity was evaluated, the tight junction-related gene levels, including *ZO-1*, *occludin*, and *claudin-1*, significantly increased when treated with BML284 and significantly decreased with ICG001 treatment, indicating that activation of Wnt/β-catenin signaling can help maintain epithelial integrity in porcine organoid monolayers (Fig. 7H through J). The TEER was also monitored to assess the impacts of Wnt/β-catenin signaling on epithelial barrier integrity. As expected, activation of the Wnt/β-catenin signaling pathway increased the TEER, while inhibition of the Wnt/β-catenin signaling pathway reduced the TEER (Fig. 7K; Fig. S3A and B), which, in turn, correspondingly promoted or inhibited early PDCoV infection (Fig. 7L; Fig. S3C).

Importantly, activation of the Wnt/β-catenin signaling pathway by treatment with BML284 increased porcine ISC renewal, accelerated the renewal, and replenished the shedding of PDCoV-infected intestinal epithelial cells in the context of PDCoV infection. This was evidenced by accumulated β-catenin, increased Wnt/β-catenin-related genes, tight junction-related genes, and TEER. Conversely, suppression of the Wnt/β-catenin signaling pathway by treatment with ICG001 following PDCoV infection decreased Wnt/β-catenin-related genes, tight junction-related genes, and TEER (Fig. 7M through W). In addition, IFA results revealed that ICG001 treatment reduced β-catenin accumulation in chicken intestinal organoid monolayers. Furthermore, PDCoV + ICG001 treatment promoted β-catenin accumulation and significantly increased the expression of Lgr5, cyclin D1, and c-Myc (Wnt target genes), BMI1 (ISCs), and PCNA (TA cells) (Fig. S3D through I). Collectively, these data suggest that ‌the‌ Wnt/β-catenin signaling pathway promotes the self-renewal of intestinal stem cells, accelerates epithelial renewal, compensates for the shedding of PDCoV-infected intestinal epithelial cells, and maintains the intestinal barrier integrity, thereby enhancing resistance to PDCoV infection.

## DISCUSSION

Typically, the life cycle of a virus in the host cell includes adsorption, invasion, viral nucleic acid release and replication, nucleocapsid assembly, and maturation, at which point the complete infectious progeny virus is released from the original cells (43). Whereas critical for cross-species transmission is the successful replication of a virus in a new host, and as previously mentioned, this has already occurred for PDCoV (44, 45). PDCoV, which originated from a rare spillover event in birds (46), is the only member of the *Deltacoronavirus* genus that can infect mammals. Unlike infection in natural hosts, PDCoV infection in unnatural hosts generally causes only mild or no symptoms and, to date, has not caused widespread infection. For example, chickens and turkey poults can be experimentally infected with PDCoV but show only mild to moderate symptoms and no lesions on necropsy (16, 21). However, the potential for transmission of PDCoV to, and the replication efficiency in, poultry has not been well studied.

Because PDCoV mainly infects intestinal epithelial cells, we used chicken and porcine intestinal organoid monolayers and found that though PDCoV infected the chicken intestinal organoid monolayers, it did not replicate; in porcine intestinal organoid monolayers, PDCoV did establish a productive infection. This result is consistent with the finding that no release of infectious particles was detected at any of the tested time points in PDCoV-infected DF-1 cells (19). Furthermore, we carried out a comparative PDCoV challenge experiment in chickens and piglets, and infected piglets, but not chickens, developed severe watery diarrhea and vomiting and exhibited obvious pathological lesions. Other reports show that PDCoV-inoculated chickens showed varying degrees of intestinal pathology/diarrhea symptoms and fecal viral RNA shedding (16, 21). There are several potential reasons for these discrepancies across studies. Firstly, PDCoV exhibits stronger host specificity for pigs, which could be an important reason for the differences in infection symptoms. Secondly, differences in chicken breeds and PDCoV strains could be a factor. Furthermore, multiple factors, such as the duration of infection exposure, diet, environment, and microbiota, may contribute to potential reasons for these discrepancies. Liang et al. found that PDCoV RNA could be detected in multiple organs (lung, kidney, jejunum, cecum, and rectum) and intestinal contents of PDCoV-inoculated chickens until 17 days post-inoculation, but no viral RNA shedding in the intestinal contents and no viral RNA in the jejunum and rectum were detected in PDCoV-inoculated chickens at 3 dpi, and no PDCoV RNA was detected in the duodenum and ileum at 5 dpi (21). When a virus is unable to completely hijack the host cell, resulting in negligible or no infectious virus and the absence of obvious cytopathic effect, the term used is abortive infection (47–51). Our results demonstrate that in chickens, PDCoV infection is aborted, but the potential for successful pig-to-chicken transmission is unknown.

The intestinal barrier (physical, chemical, and immune) is the first line of defense against environmental aggression and pathogens (25, 52–54). Tight junctions are essential for the proper function of the intestinal epithelium’s physical barrier. Deficient tight junctions lead to barrier disruption and increased intestinal epithelial permeability, one cause of diarrhea (55). Porcine epidemic diarrhea virus (PEDV)-inoculated piglets developed severe diarrhea and, upon necropsy, showed extensive disruption of tight junctions (56). Similarly, we found that PDCoV infection disrupts the tight junctions in the intestinal barrier of pigs and causes severe diarrhea. Another important physical barrier that protects the intestinal epithelium from pathogens is the intestinal mucus layer. Mucus is synthesized and secreted by goblet cells (52, 57). We found that in pigs, PDCoV infection significantly reduced the number of goblet cells and *MUC2* expression. In PDCoV-infected chickens, goblet cell numbers were increased over controls as was *MUC2* expression. This may be one reason for the insensitivity of chickens to PDCoV.

A growing body of research shows that the Wnt/β-catenin pathway plays an indispensable role in the proliferation and differentiation of ISCs (37, 58, 59). Previous studies have reported that both *Salmonella* and transmissible gastroenteritis virus (TGEV) infection activate the Wnt/β-catenin pathway, resulting in an increase in the number of stem cells and proliferative cells, which in turn promotes intestinal epithelium regeneration (60, 61). Interleukin (IL)-6- and tumor necrosis factor (TNF)-induced inflammatory responses can promote stem cell self-renewal and differentiation to repair the damaged epithelium (62, 63). In our study, we found that in piglets, PDCoV infection activated the innate immunity, induced IL-6 and TNF expression, and activated the Wnt/β-catenin signaling pathway, stimulating the self-renewal of ISCs both in intestinal organoids and jejunum of newborn piglets. In our porcine organoid monolayers and Transwell system, BML284 treatment (activator of the Wnt/β-catenin pathway) resulted in increased ISC self-renewal and accelerated the renewal of intestinal epithelial cells in the context of PDCoV infection, as shown by accumulated β-catenin, increased levels of Wnt/β-catenin-related genes, tight junction-related genes, and TEER. Conversely, ICG001 (inhibitor of the Wnt/β-catenin pathway) resulted in lower levels of Wnt/β-catenin-related genes, tight junction-related genes, and TEER. However, PDCoV infection did not result in enough new cells to be generated from ISCs to cope with the damage caused by the infection. In contrast, in chicken organoids, abortive infection of PDCoV triggered activation of the Wnt/β-catenin pathway but did not induce innate immunity and inflammatory responses. In addition, Boley et al. were unable to observe a positive signal in the tissues from PDCoV-infected chicks but found PDCoV antigen in numerous epithelial cells from infected poults, which were sloughed off and remained in the lumen of infected poults (16). It could be seen that ISC renewal and epithelial regeneration were substantial, allowing repair of the limited damage induced by PDCoV infection.

**Fig 8:**
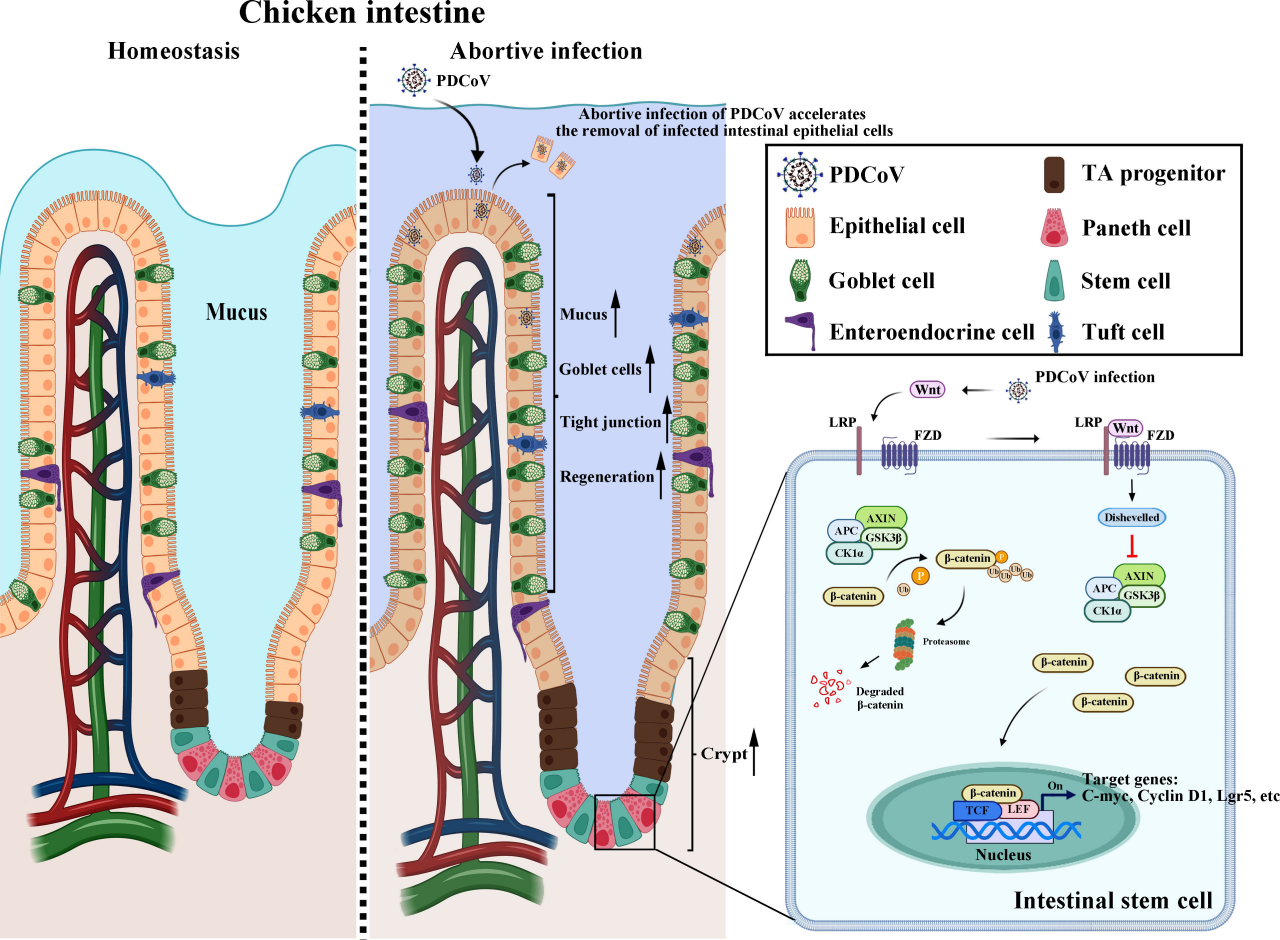
Schematic diagram of abortive infection induced intestinal epithelial regeneration via the Wnt/β-catenin pathway in PDCoV-infected chicken intestine.

In summary, we show that PDCoV has the potential for transmission between pigs and chickens. PDCoV is infective to chickens but fails to replicate *ex vivo* and *in vivo*. Abortive infection of PDCoV protects the chicken intestinal physical barrier (mucus layer and tight junctions) and stimulates the Wnt/β-catenin pathway, triggering self-renewal and differentiation of chicken ISCs, which accelerates the regeneration of chicken epithelium and the intestinal damage repair (Fig. 8). Although the chicken is regarded as a dead-end host for PDCoV, the possibility of PDCoV evolving with the ability to infect and spread efficiently in chickens cannot be ruled out. Therefore, close surveillance for PDCoV in chickens is prudent to avoid virus spillover and epidemic outbreak.

## MATERIALS AND METHODS

### Virus

PDCoV (CHN-GD16-05) was kindly provided by Professor Zhenhai Chen from Yangzhou University.

### Isolation and culture of chicken and porcine intestinal organoids

Intestinal crypts were isolated from the duodenum, jejunum, ileum, and cecum of 18-day-old specific pathogen-free (SPF) chicken embryos and from the jejunum of newborn piglets. Crypts were cultured in a 3D organoid culture system with the support of Matrigel matrix (Corning, USA) and IntestiCult Organoid Growth Medium (StemCell Technologies, Canada) following previously described protocols (64, 65). Fresh growth medium was changed every 2–3 days, and the morphology of organoids was observed daily by light microscopy.

Well-developed chicken and porcine organoids were used to develop planar (two-dimensional) intestinal organoid monolayers. The organoids were collected from Matrigel after washing twice with ice-cold Dulbecco's modified Eagle medium (DMEM)/F12 medium and dissociation with TrypLE Express (GIBCO, USA) for 10 min at 37°C. Cells were then seeded into 48-well plates (Corning) or Transwell culture plates (Corning) and cultivated in a humidified 5% CO_2_ atmosphere at 37°C. The intestinal organoid monolayers reached confluence after 3–4 days.

### Organoid fixation and embedding for hematoxylin and eosin staining

Organoids were collected and fixed in 4% paraformaldehyde at 4°C for 1 h. Cells were pelleted then resuspended in cold 30% sucrose and incubated for 3 h at 4°C. Organoids were pelleted again then suspended in approximately 50 µL of supernatant, then layered on top of 450 µL of optimal cutting temperature embedding compound in the Tissue-Tek cryomold (Sakura). Organoids were centrifuged to the bottom of the embedding mold and prepared for freezing and cryosection. Frozen sections were subjected to H&E staining using an H&E Stain Kit (Solarbio, Beijing, China) according to the manufacturer’s protocol.

### Indirect immunofluorescence

The cultured intestinal organoids and organoid monolayers were fixed with 4% paraformaldehyde for 20 min at room temperature, permeabilized with 0.1% Triton X-100 in phosphate-buffered saline (PBS) for 10 min, then blocked with 5% bovine serum albumin (BSA) for 1 h at 37°C. Organoids were then incubated with primary antibody (anti-E-cadherin, anti-Ki67, anti-Lyz, anti-Lgr5, or anti-PDCoV-N at 1:100 or 1:200) for 12 h at 4°C, then with the appropriate second antibody (Dylight 488, 549, or 649 goat anti-mouse IgG H&L or Dylight 649 goat anti-rabbit IgG H&L at 1:200) for 30 min, and followed by incubating 1 µg/mL 4′,6-diamidino-2-phenylindole (DAPI) for 10 min at room temperature. The samples were visualized and imaged using a Leica TCS SP8 STED laser scanning confocal microscope (Leica, Germany) and analyzed using Leica LAS AF Lite (Leica).

### Virus infection

Confluent organoid monolayers were washed and incubated with PDCoV in a humidified 5% CO_2_ atmosphere for 1 h at 37°C. Unattached virus was then removed by washing three times with PBS, then the organoid monolayers, cultured in IntestiCult Organoid Growth Medium containing 8 µg/mL of trypsin, were either collected for RNA extraction or stained at various times post-infection.

### RNA extraction and quantitative real-time PCR

Total RNA extraction from organoid monolayers or homogenized tissues was conducted using RNAiso reagent (TaKaRa, Dalian, China) according to the manufacturer’s protocol. The quality and concentration of RNA were measured by Nanodrop (Thermo Scientific, USA), and 1 µg RNA from each sample was subjected to reverse transcription using HiScript II Q RT SuperMix (Vazyme, Nanjing, China). Gene expression levels were quantified by RT-qPCR using TB Green Premix Ex Taq II (TaKaRa) in the Applied Biosystems 7500 Real-Time PCR system (Applied Biosystems, USA). *GAPDH* served as the reference, and the relative gene expression was calculated with the 2^−ΔΔCt^ method from three biological replicates. Primer-BLAST (https://www.ncbi.nlm.nih.gov/tools/primer-blast/index.cgi) was used for primer design, and primer efficiency was verified for each primer pair by melt curve analysis. Primers for specific genes are listed in Table S1.

### Plaque assay

Confluent ST cells were incubated with serial 10-fold dilutions of supernatant from mock- or PDCoV-infected organoid monolayers for 2 h at 37°C. The supernatants were then removed, and the cells were overlaid with 0.7% low-melting-point agarose in DMEM/trypsin (10 µg/mL) and incubated at 37°C for 72 h or until the appearance of plaques. The cells were stained with 1% crystal violet in methanol to visualize plaques.

### Transepithelial electrical resistance

Well-developed intestinal organoids were dissociated into single cells, and 2.5 × 10^4^ cells were then seeded on 24-well transparent Transwell inserts (0.4 µm pore size, 6.5 mm diameter; Corning). The TEER of organoid monolayers with or without PDCoV infection was measured using a Millicell ERS‐2 (Merck Millipore, USA) as previously described (66): resistance of a unit area = (total resistance − blank resistance) (Ω)  × effective membrane area (cm^2^).

### Establishment of the monolayer enteroid/ST cell co-culture system

Dissociated intestinal organoids (2.5 × 10^4^ cells) were seeded into the upper chamber of 24-well Transwell inserts and cultured until a TEER of >1,000 Ω ·cm^2^ was obtained. The organoid monolayers were infected with PDCoV for 1 h at 37°C, then washed three times with PBS to remove the unattached virus. Subsequently, the upper chambers of the inserts were transferred to lower chambers containing confluent ST cells and co-cultured for 24 h. Cytopathic effects of ST cells were observed under a light microscope. ST cells were collected for RNA isolation.

### 5-Ethynyl-20-deoxyuridine (EdU) staining

EdU staining was performed using an EDU kit (RiboBio, Guangzhou, China) according to the manufacturer’s instructions. Briefly, the intestinal organoids or organoid monolayers were incubated with 50 µM EdU medium for 2 h before fixation. After fixation with 4% paraformaldehyde and permeabilization with 0.5% Triton X-100, the cells were incubated with 1× Apollo reaction cocktail for 30 min at room temperature. Subsequently, nuclei were stained with 1 µg/mL DAPI for 10 min at room temperature. The cells were visualized and imaged under a Leica TCS SP8 STED laser scanning confocal microscope (Leica) and analyzed using Leica LAS AF Lite (Leica).

### Animal experiments

Twenty 3-day-old SPF chickens were randomly divided into two groups: mock and PDCoV. All chickens had free access to water and feed throughout the experiment. Eight healthy Duroc × Landrace × Large White neonatal 0-day-old piglets with similar weights were selected from the same litter in a commercial farm. The herd tested negative for antibodies to PDCoV, TGEV, PEDV, PRRSV, PRCV, and PCV2. The piglets were randomly divided into two groups, mock and PDCoV, and fed 10 mL milk every 3 h. Each group of chickens and pigs was housed in separate incubators that were supplied with a filtered intake and exhaust air. Chicken-PDCoV and pig-PDCoV groups were orogastrically inoculated with 1 × 10^6^ TCID_50_ of PDCoV CHN-GD16-05, and the uninfected control chickens and piglets (mock) were orally administered an equal volume of PBS. All chickens and pigs were sacrificed and necropsied when PDCoV-infected piglets showed obvious clinical signs of vomiting, severe diarrhea, and lethargy at 48 hpi. Samples from small intestines were collected, stored at −80°C until used for RT-qPCR and Western blotting, or embedded in paraffin and cut into 4 µm sections for H&E, PAS, and immunohistochemical staining, or cut into 1 mm^3^ pieces for scanning electron microscopy (SEM) and transmission electron microscopy (TEM).

### Histological and immunohistochemical staining

Paraffin-embedded intestinal segments were serially sectioned into 5 µm sections. Sections were dewaxed in xylene then rehydrated in a series of decreasing concentrations of ethanol. Sections were then stained with hematoxylin and eosin by using an H&E Stain Kit (Solarbio) according to the manufacturer’s protocol. Goblet cells were stained using a PAS Stain Kit (Solarbio) according to manufacturer’s instructions.

For the immunochemistry assay, sections were dewaxed and rehydrated as described above. Antigen retrieval was performed by incubating sections in 10 mM citrate buffer (pH 6.0) in a decloaking chamber for 30 min at 95°C. The sections were blocked with 5% BSA for 1 h at 37°C then incubated with primary antibodies (1:100, anti-PDCoV-N, anti-MUC, anti-c-myc, and anti-PCNA) overnight at 4°C. A SABC-POD kit (rabbit or mouse IgG) and a peroxidase substrate kit (both from BOSTER, Wuhan, China) were used for amplification and visualization of signal. The sections were counterstained with hematoxylin and mounted in neutral resin sealing.

### TEM and SEM

Jejunum segments were cut into approximately 1 mm^3^ for TEM and SEM. For TEM, the samples were fixed in 2.5% glutaraldehyde for 24 h at 4°C, post-fixed in 1% OsO_4_ for 1 h at room temperature, and dehydrated through a series of increasing ethanol and acetone concentrations. Finally, the specimens were embedded in epoxy resin and sectioned for observation by transmission electron microscope (Hitachi H-600; Hitachi, Ltd., Tokyo, Japan). For SEM, the jejunum samples were fixed with 2.5% glutaraldehyde for 2 h at 4°C, then dehydrated through a graded series of ethanol concentrations. After dehydration in a freeze dryer, the samples were sputter coated with gold and then visualized by a scanning electron microscope (GeminiSEM 300; Carl Zeiss, Germany).

### Statistical analysis

Data are presented as means ± standard deviation from three independent experiments. Outliers were determined by Grubbs’ test (alpha = 0.05) and excluded from the analyses. Student’s *t*-test was used to compare the significant differences between mock- and PDCoV-infected groups, and one-way analysis of variance was used for comparisons of more than two groups using GraphPad Prism version 6 software. Differences were considered statistically significant at *P <  0.05, **P < 0.01, with ns defined as not significant.

## Data Availability

All data in this study are presented in the article and its supplemental material.

## SUPPLEMENTAL MATERIAL

Supplemental material jvi.00137-25-s0001.docx Figures S1 to S3; Table S1.

ASM does not own the copyrights to Supplemental Material that may be linked to, or accessed through, an article. The authors have granted ASM a non-exclusive, world-wide license to publish the Supplemental Material files. Please contact the corresponding author directly for reuse.
