# Supplementary material for: Abortive PDCoV infection triggers Wnt/β-catenin pathway activation, enhancing intestinal stem cell self-renewal and promoting chicken resistance
Source: J Virol. 2025 Mar 26;99(4):e00137-25. doi: 10.1128/jvi.00137-25 (PMC11998530; doi:10.1128/jvi.00137-25)
Supplement: Supplemental material — Figures S1 to S3; Table S1. [file jvi.00137-25-s0001.docx]

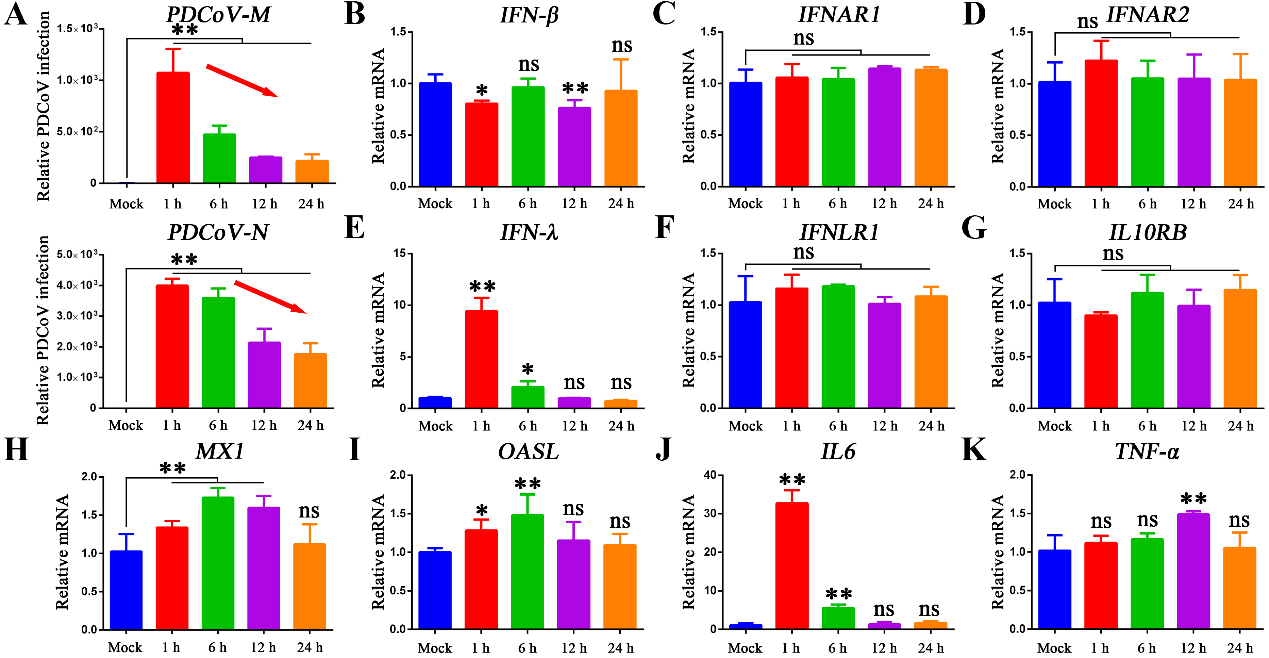


**Fig. S1** (**A**) Viral replication in chicken intestinal 2D monolayer organoids was quantified by RT-qPCR (*PDCoV-M*, *PDCoV-N*) at different time points. (**B-K**) mRNA levels of interferons and their respective cellular receptors (*IFN-β*, *IFNAR1*, *IFNAR2*, *IFN-λ*, *IFNLR1*, and *IL10RB*), interferon-stimulated genes (*MX1* and *OASL*), and proinflammatory factors (*IL6* and *TNF-α*) from PDCoV-infected chicken enteroids at different time points. * *p* < 0.05, ** *p* < 0.01, ns not significance.


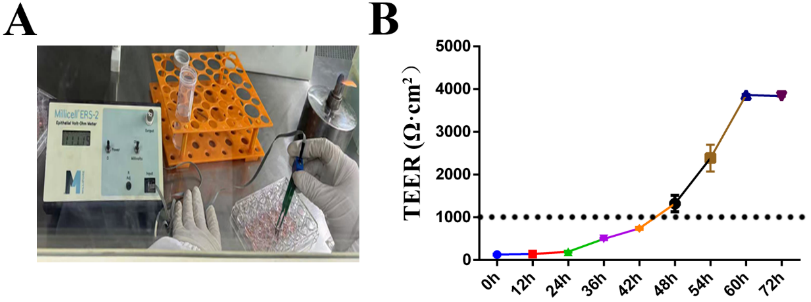


**Fig. S2** (**A**) TEER of chicken organoid monolayers reaching a plateau at 60 h. (**B**) TEER of chicken organoid monolayers over 72 h. Resistance of a unit area = (Total resistance − blank resistance) (Ω) × Effective membrane area (cm^2^).


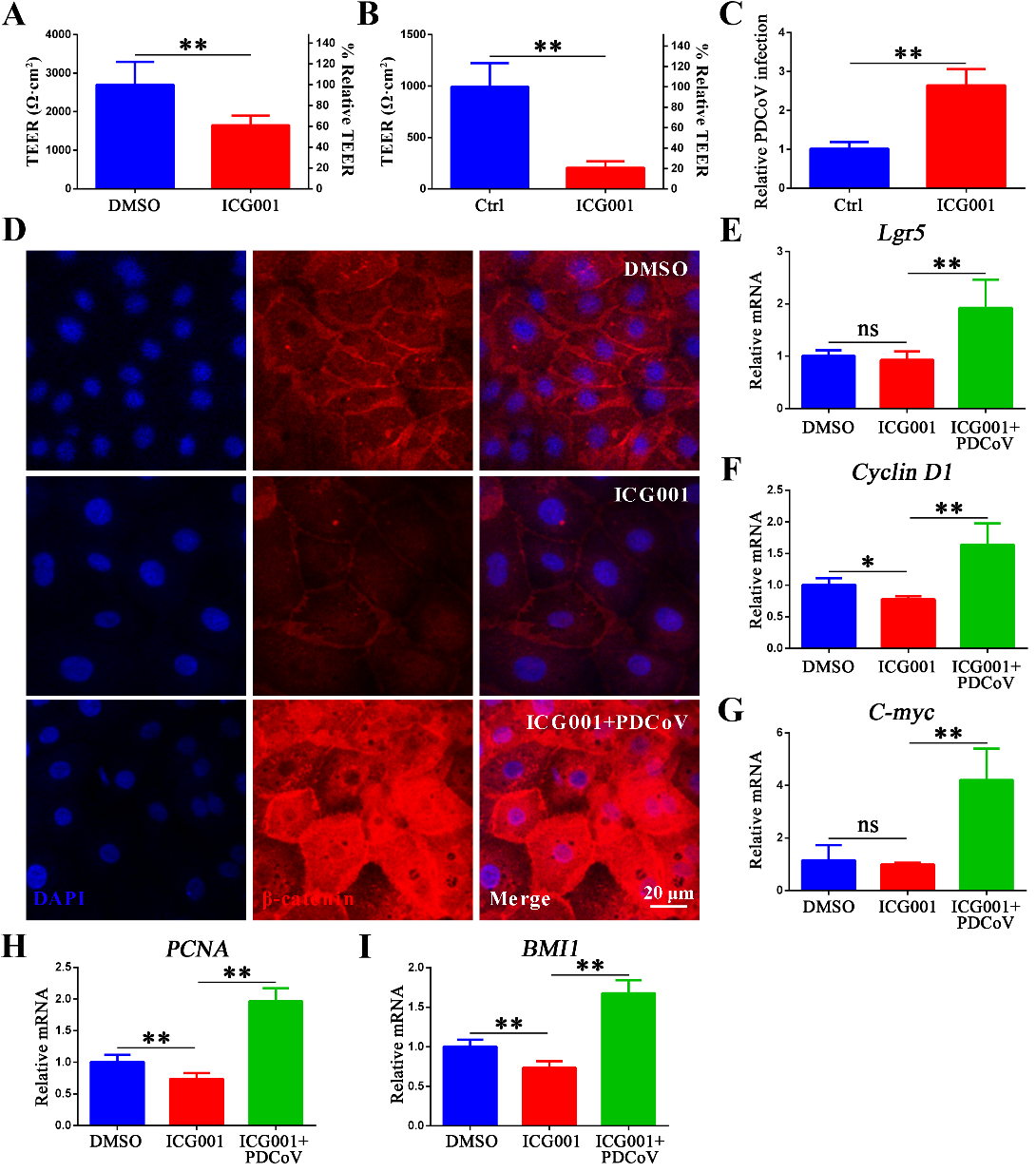


**Fig. S3** (**A**) TEER values were calculated from ICG001 treated chicken intestinal organoid monolayers. (**B**) TEER values were calculated from PDCoV-infected cells from Figure A at 24 hpi. (**C**) Viral replication in chicken intestinal organoid monolayers from Figure B was quantified by RT-qPCR. (**D**) The nuclear translocation of β-catenin was observed in chicken intestinal organoid monolayers by confocal microscopy. Scale bar = 20 μm. (**E-I**) mRNA levels of *Lgr5*, *Cyclin D1*, *c-Myc*, *PCNA*, and *BMI1* in chicken intestinal organoid monolayers. * *p* < 0.05, ** *p* < 0.01, ns not significance.

**Table S1 Primer sequences for RT-qPCR**

| Primers | Sequence (5′ - 3′) | Accession |
| --- | --- | --- |
| PDCoV-N | F: CCCAGCTCAAGGTTTCAGAG | KY363868.1 |
|  | R: ATTGGCACCAGTGCGAGACC |  |
| PDCoV-M | F: TCCAATGGGTACATGGAGGT | KY363868.1 |
|  | R: TGGATCGTTGTTTGATTCCA |  |
| Pig-GAPDH | F: CACAGTCAAGGCGGAGAAC | NM_001206359.1 |
|  | R: CGTAGCACCAGCATCACC |  |
| Pig-IFN-β | F: TGCATCCTCCAAATCGCTCT | NM_001003923.1 |
|  | R: ATTGAGGAGTCCCAGGCAAC |  |
| Pig-IFNAR1 | F: AGAGCAGCAGTGAATCCGTC | NM_213772.1 |
|  | R: CCAATTATCCATCCCTGTTATTTGA |  |
| Pig-IFNAR2 | F: CCTGCTTGGAAGCTGATGTG | NM_001204775.2 |
|  | R: CAAACACGAGGCTGATGTGC |  |
| Pig-IFN-λ | F: CCACGTCGAACTTCAGGCTT | NM_001142837.1 |
|  | R: ATGTGCAAGTCTCCACTGGT |  |
| Pig-IFNLR1 | F: TGGCCTATCAAAGCTCAGCAA | XM_021095537.1 |
|  | R: GAGGATCTCCTCTGTCCGGT |  |
| Pig-IL10RB | F: TCGGAACAAAACCGGGGAAT | NM_213771.1 |
|  | R: AAATGCACCGCAGCAAGATG |  |
| Pig-MX1 | F: TCCAGCCACATCCCTCT | NM_214061.2 |
|  | R: GCCAGTCGTATTGGTCCTT |  |
| Pig-OASL | F: GTGTGGTTGGTCGAGGAAGT | NM_001031790.1 |
|  | R: TGTAGGCAGGCATGATGGTG |  |
| Pig-IL6 | F: CCTCGGCAAAATCTCTGCAA | NM_214399.1 |
|  | R: TGAAACTCCACAAGACCGGT |  |
| Pig-TNF-α | F: CGACTCAGTGCCGAGATCAA | NM_214022.1 |
|  | R: CCTGCCCAGATTCAGCAAAG |  |
| Pig-MUC2 | F: ACGACTTTGACGGACACTGCT | XM_002347185.2 |
|  | R: AGGGGACGTTCTCGGTGAT |  |
| Pig-ZO-1 | F: AGCCCGAGGCGTGTTT | XM_021098856.1 |
|  | R: GGTGGGAGGATGCTGTTG |  |
| Pig-Occludin | F: ATCAACAAAGGCAACTCT | NM_001163647.2 |
|  | R: GCAGCAGCCATGTACTCT |  |
| Pig-Claudin1 | F: ACCCCAGTCAATGCCAGATA | NM_001244539.1 |
|  | R: GGCGAAGGTTTTGGATAGG |  |
| Pig-Wnt3a | F: GAGTGCCAACACCAGTTCC | XM_021083340.1 |
|  | R: AGTCACAGCGAAGGCAACTC |  |
| Pig-Lrp5 | F: CGACGTGTCCACTAACCACA | XM_021082723.1 |
|  | R: GTCGGCGTAGTCAATGGTGA |  |
| Pig-β-catenin | F: GACCATGCCATGATTGGACCTGAG | NM_001315760.1 |
|  | R: GCCTGTCAACCTTCTCGCTGTC |  |
| Pig-TCF4 | F: TAATGGAGCAATGGGTGGTC | XM_021093592.1 |
|  | R: CCTGGTTTGGGACAAGAGAA |  |
| Pig-Lgr5 | F: CCTTGGCCCTGAACAAAATA | NM_001315762.1 |
|  | R: ATTTCTTTCCCAGGGAGTGG |  |
| Pig-Cyclin D1 | F: GCGAGGAACAGAAGTGCG | XM_021082686.1 |
|  | R: TGGAGTTGTCGGTGTAGATGC |  |
| Pig-C-myc | F: AACAACCGAAAATGCGCCAG | NM_001005154.1 |
|  | R: ACATTGTGTGTCCGCCTCTT |  |
| Pig-PCNA | F: GATTCCACCACCATGTTCGAG | NM_001291925.1 |
|  | R: GATTCCACCACCATGTTCGAG |  |
| Pig-BMI1 | F: CTTCAAGATGGCCGCTTGGC | NM_001285971.1 |
|  | R: AAGCGGGCGGAAAAGACAAT |  |
| Pig-Lyz | F: GGTCTATGATCGGTGCGAGT | NM_214392.2 |
|  | R: AACTGCTTTGGGTGTCTTGC |  |
| Chicken-GAPDH | F: TGATGGTCCACATGGCATCC | NM_204305.1 |
|  | R: GGGAACAGAACTGGCCTCTC |  |
| Chicken-IFN-β | F: TTGCCCACAACAAGACGTGA | NM_001024836.2 |
|  | R: ATCTGGTTGAGGAGGCTGTG |  |
| Chicken-IFNAR1 | F: CACATCCGCATAAGGGCTGA | NM_204859.2 |
|  | R: AGCCACATTTTTCGAACCTGAT |  |
| Chicken-IFNAR2 | F: ATTCCATCCCATCAGCCTGG | NM_001397696.1 |
|  | R: TGGCATCGCTTTCACTGTCA |  |
| Chicken-IFN-λ | F: GTACCAGTTCCCTGCACCTT | NM_001128496.1 |
|  | R: ACCGACAGCTCAGCTATGTC |  |
| Chicken-IFNLR1 | F: CCTTCTGACAAGTGCCTCCA | NM_001389541.2 |
|  | R: GCAAAAAGTTGGTCCAGCAAT |  |
| Chicken-IL10RB | F: AGGACGGAGTGGGAAGATGA | NM_204857.2 |
|  | R: CGGTAAATCCACGAGCCGTA |  |
| Chicken-MX1 | F: AGCGCTGAAAATGGCTCAAG | NM_204609.2 |
|  | R: GTTGGGTGTGTGCTTTGTGT |  |
| Chicken-OASL | F: CACGGCCTCTTCTACGACAT | NM_001397447.1 |
|  | R: AGCTCCTTGGTCTCGTAGGT |  |
| Chicken-IL6 | F: CGGCAGATGGTGATAAATCC | NM_204628.2 |
|  | R: CCCTCACGGTCTTCTCCATA |  |
| Chicken-TNF-α | F: GGCAGCTGTGGTGCAAATAA | NM_204267.2 |
|  | R: CACAACACGGCTTCAGCATC |  |
| Chicken-MUC2 | F: ATTGTGGTAACACCAACATTCATC | XM_040673077.2 |
|  | R: CTTTATAATGTCAGCACCAACTTCTC |  |
| Chicken-ZO-1 | F: GACAGCCAACAAGGCAAGTG | XM_046925214.1 |
|  | R: TGCATCCCATCCAGGTCCTA |  |
| Chicken-Occludin | F: ACAGCCCTCAATACCAGGATGTG | NM_205128.1 |
|  | R: ACCATGCGCTTGATGTGGAA |  |
| Chicken-Claudin1 | F: AGGTGTACGACTCGCTGCTT | NM_001013611.2 |
|  | R: GGGCATTTTTGGGGTAGCCT |  |
| Chicken-Wnt3a | F: CCGGGGTTGGGTAGAAACTC | NM_001081696.2 |
|  | R: GGTCACAGCCATCAATCCCA |  |
| Chicken-Lrp5 | F: TGGCTGTAGACTGGATGGGA | NM_001012897.2 |
|  | R: ACAGCCGTTGGTCTGCATAA |  |
| Chicken-β-catenin | F: TCTCACATCACCGTGAAGGC | NM_205081.3 |
|  | R: TGGAGCAGACTGACAACACC |  |
| Chicken-TCF4 | F: TTCGGAAGGTTCCTCCGGG | XM_046937299.1 |
|  | R: ATGCGAGTGCTTTCCCCAAC |  |
| Chicken-Lgr5 | F: TACGTCTTGCAGGAAATGGCT | XM_046909876.1 |
|  | R: GGAACCTGGCGTAGTTGGTTA |  |
| Chicken-Cyclin D1 | F: GACCCGACGAGTTACTGCAA | NM_001396513.1 |
|  | R: GAGCCACAAAAGTCTGAGCA |  |
| Chicken-C-myc | F: ATCGACCCCTCGGTGGTCTT | NM_001030952.2 |
|  | R: GGCTGGGTATTCCACCTTGG |  |
| Chicken-PCNA | F: AATGCGGATACGTTGGCTCT | NM_204170.3 |
|  | R: CACCAATGTGGCTGAGGTCT |  |
| Chicken-BMI1 | F: ATCGTGCGGTACTTGGAGAC | NM_001007988.3 |
|  | R: TTTGAAAAGGCCCGGTACGA |  |
| Chicken-Lyz | F: TGAAGCGTCACGGACTTGAT | NM_205281.2 |
|  | R: TCTCGAATTTTGCGGCACAC |  |
